# The Spectrum of Pathogens Associated with Infections in African Children with Severe Acute Malnutrition: A Scoping Review

**DOI:** 10.3390/tropicalmed9100230

**Published:** 2024-10-06

**Authors:** Bassey Ekeng, Olufunke Adedokun, Vivien Otu, Stella Chukwuma, Agatha Okah, Osamagbe Asemota, Ubokobong Eshiet, Usenobong Akpan, Rosa Nwagboso, Eti Ebiekpi, Emmanuella Umoren, Edet Usun

**Affiliations:** 1 Department of Medical Microbiology and Parasitology, University of Calabar Teaching Hospital, Calabar 540271, Nigeria; 2 Department of Paediatrics, University of Calabar Teaching Hospital, Calabar 540271, Nigeria; 3 Department of Medical Microbiology, College of Medicine, Enugu State University of Science and Technology, Enugu 400283, Nigeria; 4 Department of Paediatrics, University Hospitals Coventry and Warwickshire, NHS Trust, Coventry CV2 2DX, UK; 5 Department of Paediatrics, University of Uyo Teaching Hospital, Uyo 520261, Nigeria; 6 Department of Family Medicine, University of Calabar Teaching Hospital, Calabar 540271, Nigeria; 7 Department of Community Medicine, University of Uyo Teaching Hospital, Uyo 520261, Nigeria; 8 Department of Paediatrics, Leeds Teaching Hospital Trust, Leeds LS1 3EX, UK

**Keywords:** severe acute malnutrition, tuberculosis, HIV/AIDS, immunocompromised, Africa

## Abstract

Understanding the interplay between infections and severe acute malnutrition is critical in attaining good clinical outcomes when managing malnourished children. However, review studies describing the profile of the associated pathogens in the malnourished African paediatric population are sparse in the literature. We aimed to identify the spectrum of pathogens from studies reporting infections in severely malnourished African children, as well as the antibiotic resistance pattern and clinical outcomes. A systematic literature review of the PubMed database was conducted following PRISMA guidelines from January 2001 to June 2024. The search algorithm was ((marasmus) OR (kwashiorkor) OR (severe acute malnutrition) OR (protein energy malnutrition)) AND (Africa). For a more comprehensive retrieval, an additional search algorithm was deployed: ((HIV) OR (tuberculosis)) AND (severe acute malnutrition). We included 60 studies conducted between 2001 and 2024. Most of the studies were from East Africa (n = 45, 75%) and Southern Africa (n = 5, 8.3%). A total of 5845 pathogens were identified comprising 2007 viruses, 2275 bacteria, 1444 parasites, and 119 fungal pathogens. The predominant pathogens were HIV, *Mycobacterium tuberculosis*, and malaria parasites accounting for 33.8%, 30%, and 24.2% of pathogens identified. Antibiotic susceptibility testing was documented in only three studies. Fatality rates were reported in 45 studies and ranged from 2% to 56% regardless of the category of pathogen. This review affirms the deleterious effect of infections in malnourished patients and suggests a gross underdiagnosis as studies were found from only 17 (31.5%) African countries. Moreover, data on fungal infections in severely malnourished African children were nearly absent despite this population being at risk. Thus, there is an urgent need to prioritize research investigating African children with severe acute malnutrition for fungal infections besides other pathogens and improve the availability of diagnostic tools and the optimized usage of antibiotics through the implementation of antimicrobial stewardship programmes.

## 1. Introduction

Severe acute malnutrition (SAM) is a significant global health issue, defined by the World Health Organization (WHO) and the United Nations International Children’s Emergency Fund (UNICEF) based on specific criteria: a weight-for-height z-score (WHZ) below −3, a mid-upper arm circumference (MUAC) under 115 mm, or the presence of nutritional oedema [1]. According to UNICEF (2022), 45 million children under five were impacted by wasting, with 13.7 million categorized as severely wasted [2]. The burden of SAM is predominantly concentrated in South Asia and sub-Saharan Africa, where factors such as drought, armed conflict, poverty, food insecurity, inadequate healthcare infrastructure, and socioeconomic instability exacerbate the prevalence of malnutrition [3]. Despite global efforts, the continent continues to struggle with high rates of undernutrition, micronutrient deficiencies, obesity, and non-communicable diseases. According to a recent report by United Nations agencies, nearly 282 million people in Africa, or about 20% of the population, were undernourished in 2022 [4]. Malnutrition rates vary significantly across African regions, with sub-Saharan Africa bearing the heaviest burden. In Eastern Africa, countries such as Ethiopia, South Sudan, and Somalia have some of the highest stunting rates, often exceeding 30% [5].

The role of climate change in exacerbating SAM is particularly concerning. The Intergovernmental Panel on Climate Change (IPCC) reports that global warming is intensifying food insecurity, especially in tropical regions where 95% of malnourished individuals reside [6]. Rising temperatures and more frequent extreme weather events, such as droughts, lead to reduced agricultural productivity, directly contributing to increased malnutrition rates. The interconnectedness of climate change and food security highlights the need for comprehensive approaches that address environmental factors in the fight against malnutrition.

SAM leads to physical wasting and severely compromises the immune system, increasing susceptibility to infections. These infections—whether bacterial, viral, parasitic, or fungal—tend to be more frequent and severe in SAM patients, further elevating metabolic demands and depleting already scarce nutrient reserves. This creates a vicious cycle where malnutrition and infection exacerbate each other, complicating recovery and worsening health outcomes [7]. Infectious diseases pose significant health risks, particularly in regions afflicted by high rates of malnutrition, such as sub-Saharan Africa. The widespread prevalence of malnutrition and opportunistic infections in sub-Saharan Africa underscores the formidable health challenges facing the region. The immune dysfunction associated with SAM can lead to a higher risk of morbidity and mortality from common childhood illnesses such as diarrhoea and pneumonia [7]. SAM is a major public health issue in Africa, affecting millions of people, particularly children under five [8]. However, large-scale reviews describing the spectrum of pathogens in children with SAM are lacking in the literature, particularly for the African setting. In addition, data on the susceptibility profile of these pathogens are fragmented in the literature. Previous reviews focused on undernutrition and associated factors among HIV-infected children in sub-Saharan Africa [9], while reviews summarizing data on pathogens in African children with SAM have largely been focused on HIV [10,11]. Studies on other groups of pathogens in this at-risk group and their antimicrobial resistance pattern are lacking. Thus, the overarching aim of this review was to highlight the burden of pathogens reported in African children with SAM and the need to drive antimicrobial stewardship practices in this setting and invariably improve clinical outcomes.

## 2. Materials and Methods

### 2.1. Study Design

We conducted a scoping review of the literature adhering to the Preferred Reporting Items for Systematic Reviews and Meta-Analyses Extension for Scoping Reviews (PRISMA-ScR) guidelines [12].

### 2.2. Search Strategy

We conducted a systematic literature search of the PubMed database between January 2001 and June 2024 (BE). The search algorithm was ((marasmus) OR (kwashiorkor) OR (severe acute malnutrition) OR (protein energy malnutrition)) AND (Africa). An additional search algorithm was deployed for a more comprehensive retrieval: ((HIV) OR (tuberculosis)) AND (severe acute malnutrition). No language restrictions were applied.

### 2.3. Inclusion and Exclusion Criteria

All articles with primary data on pathogens in African children with SAM were eligible for inclusion. The inclusion of cases was based on the WHO classification of nutritional status of infants and children for SAM: weight-for-length/height or BMI-for-age < −3 SD of the median, a mid-upper arm circumference <115 mm, or bilateral pitting oedema [13]. Studies reporting infections in children with other forms of malnutrition including obese, overweight, underweight, stunting, or wasting were not eligible for inclusion. In addition, studies reporting infections in severely malnourished children outside Africa were excluded. Review articles were also excluded.

### 2.4. Selection Process

Two authors (BE and OA) initially screened titles and abstracts, focusing on studies reporting pathogens in severely malnourished African children. Selected studies were further screened, and duplicates were removed as more than one search algorithm was deployed. Full-text assessment was thereafter performed and followed by data extraction. Discrepancies in inclusion/exclusion decisions were resolved through a discussion.

### 2.5. Data Extraction

Data on study authors, study location, (country and region in Africa), study period, study design, age range, pathogens, investigation/diagnostic measures, and treatment outcomes (fatality/mortality rates) were extracted. Four authors (BE, OA, UE, and AO) performed data extraction, and any indifferences were resolved by a consensus. Descriptive statistics was used to summarize the findings.

## 3. Results

### 3.1. Search Results

**Figure 1:**
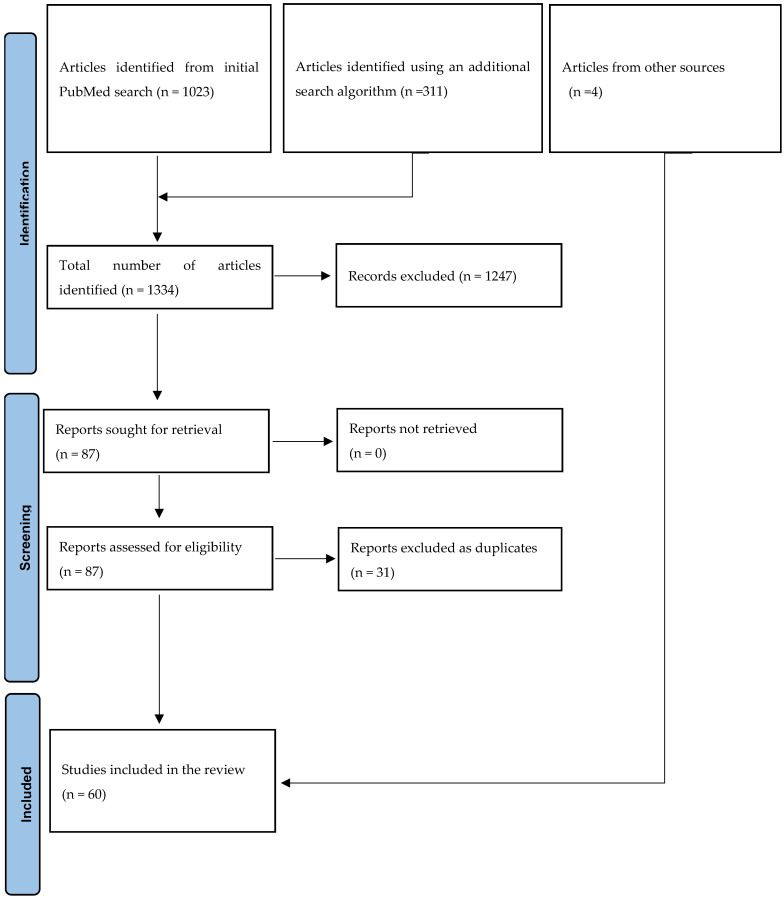
PRISMA flow diagram.

Our initial search yielded 1023 articles and 311 articles following an additional search, amounting to a total of 1334. After the selection process, 87 articles were identified as having met the inclusion criteria. Others were excluded for several reasons including a lack of information regarding this review, clinical trial studies, reviews, guidelines, studies reporting infections in moderately malnourished or underweight African children, studies from non-African paediatric populations, and studies reporting infection in adults, among others. Of the 87, 31 duplicates were removed, resulting in 56 articles. Four articles were added from other sources; thus, a total of sixty articles were included in this review, as shown in Figure 1.

### 3.2. Demographics

We included 60 studies conducted between 2001 and 2024. Data were found from only 17 (31.5%) of the 54 African countries including Ethiopia (n = 25), Uganda (n = 10), South Africa (n = 5), Malawi (n = 3), Zambia (n = 2), Mozambique (n = 2), Niger (n = 2), and Nigeria, Cameroon, Senegal, Sudan, Zimbabwe, Kenya, Sierra Leone, Democratic Republic of Congo, and Ghana (one study each). Two studies were conducted in centres located in two different countries: Kenya/Tanzania and Zambia/Zimbabwe. When stratified by regions, most studies were from East Africa (n = 45, 75%) and Southern Africa (n = 5, 8.3%).

### 3.3. Study Designs

The study designs were retrospective (48.3%, n = 29), prospective (31.7%, n = 19), cross-sectional (n = 18.3%, n = 11), and case-control (1.7%, n = 1). Fifty-nine (98.3%) were hospital-based studies and one (1.7%) was an outpatient therapeutic programme.

### 3.4. Pathogens

**Figure 2:**
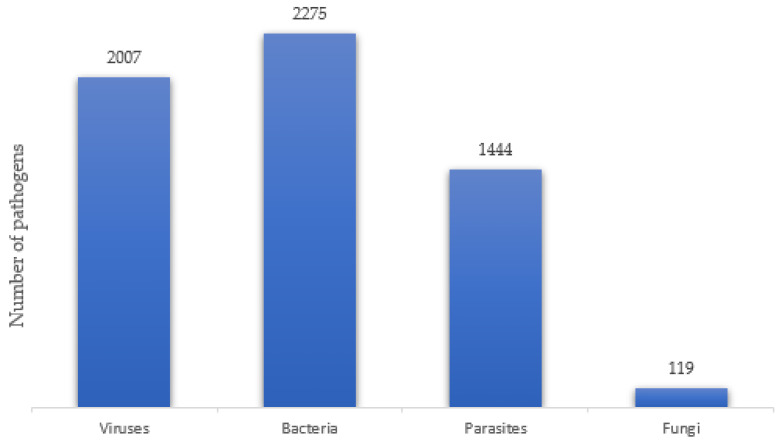
A pictorial representation of pathogens reported in severely malnourished African children.

A total of 5845 pathogens were identified. Viruses comprised 34.3% (n = 2007) and were predominantly HIV (98.5%, n = 1976), bacteria comprised 38.9% (n = 2275), mostly *Mycobacterium tuberculosis* (77.1%, n = 1753), and parasites constituted 24.7% (n = 1444), the commonest among which were malaria parasites (97.9%, n = 1414) and fungal pathogens (2.0%, n = 119). HIV infection, TB, and malaria accounted for 33.8%, 30%, and 24.2%. Figure 2 shows a snapshot of the number of pathogens identified in their respective categories. Cases of infections without a mention of associated pathogens were excluded from the analysis of pathogens identified in this review. Overall, bacteria and viruses were majorly implicated followed by parasites and fungi. Fatality rates were reported in 45 studies and ranged from 2% to 56% regardless of the category of pathogen, as shown in Table 1. A detailed summary of the findings is highlighted in Supplementary Table S1.

## 4. Discussion

SAM poses a significant public health concern, particularly affecting children under the age of five. It is characterised by extreme thinness and severe deficiencies in essential nutrients. The World Health Organization (WHO) reports that almost 16 million children globally are impacted by SAM, with a higher prevalence in sub-Saharan Africa [74]. The clinical indicators of SAM encompass substantial weight loss, low weight-for-height, and frequently, oedema. The criteria for identification include mid-upper arm circumference (MUAC) and weight-for-height Z-score evaluations [1]. Children afflicted by SAM face a significantly heightened risk of mortality due to compromised immune function stemming from malnutrition [17]. Malnutrition undermines the body’s defence against infections by compromising physical barriers such as the skin and mucous membranes, facilitating pathogen entry, and increasing infection risk. It also disrupts immune cell production and function, resulting in reduced T cell and B cell counts and activity, which are pivotal for an effective immune response [74]. In addition, SAM can incite an imbalance in cytokine production, thus increasing vulnerability to various diseases including pneumonia, diarrhoea, tuberculosis, and opportunistic infections [75]. Also, SAM may yield long-term developmental issues, affecting physical growth and cognitive development [76]. In contrast, infections can also predispose children to malnutrition with diarrheal disease following gastrointestinal infection. Cachexia and anaemia can also result from infections like HIV/AIDS and TB and nutrient deprivation resulting from parasitic infections [75]. Our review highlights over five thousand cases of opportunistic infections in malnourished children living in Africa over the past two decades, with HIV, *Mycobacterium tuberculosis*, and malaria parasites being the predominant associated pathogens. A significant proportion of these data were obtained from studies conducted in East African countries and Southern Africa, with few cases from West and North Africa, suggesting an underestimation of the burden of infectious diseases in this at-risk group.

### 4.1. Viral Infection

The predominance of HIV among pathogens reported in malnourished African children may be associated with the high burden of HIV in Africa. As of 2022, about 25.6 million people were living with HIV in Africa, accounting for more than two-thirds of the people living with HIV worldwide (WHO). Malnutrition exerts a deleterious effect on the production and functionality of immune cells, thereby diminishing the body’s capacity to combat infections such as HIV. Concomitantly, economic challenges impede access to nourishing sustenance and healthcare, exacerbating malnutrition and elevating the susceptibility to HIV infection, creating a cycle that further weakens the immune system [3].

The high prevalence of HIV among SAM children may also be linked with vertical transmission [77]. Malnourished pregnant women with HIV are more likely to have higher viral loads, increasing the risk of passing the virus to their children during pregnancy, childbirth, or breastfeeding [78]. Furthermore, in regions affected by severe poverty and malnutrition, individuals may engage in high-risk behaviours such as transactional sex to obtain food or income, increasing the risk of HIV transmission [79]. HIV worsens malnutrition by causing a loss of appetite, poor absorption of nutrients, and increasing metabolic demands. This sets in motion a challenging cycle where malnutrition worsens HIV outcomes, and HIV further deteriorates nutritional status, leading to rapid health decline [80]. This scenario is buttressed in a study aiming to ascertain 52-week mortality in children discharged from hospitals for the management of complicated SAM which was conducted in three hospitals in Zambia and Zimbabwe. Children with underlying HIV infection were observed to have an almost 4-fold higher mortality compared with children without underlying HIV infection [21]. Likewise, a South African study reported that HIV status was a significant predictor of death in children with SAM, whereby 19% of children living with HIV died compared with 3.6% of children without underlying HIV infection [14]. Similarly, a Mozambican study evaluating the adherence of malnourished children to nutritional rehabilitation programmes reported a higher prevalence of SAM among participants with underlying HIV infection [24]. Thus, besides predisposing children to malnutrition, HIV infection in malnourished children is associated with fatal clinical outcomes.

Besides HIV infection, other viruses implicated in the reviewed studies were rotavirus and adenovirus [23]. Rotavirus worsens malnutrition by reducing nutrient absorption and increasing nutrient loss, as it causes severe diarrhoea. Though commonly associated with respiratory diseases, an adenovirus infection can also present with diarrhoea, thus complicating SAM. Worthy of note is the measles virus which can cause high rates of fatalities, up to 10% in populations with severely malnourished children [Measles—PAHO/WHO|Pan American Health Organization]. The underlying mechanism remains the weakened immune system in children with SAM, hence the need to ensure vaccination and infection control measures.

### 4.2. Bacterial Infection

Next to HIV are bacterial infections presenting as TB, pneumonia, diarrheal disease, and urinary tract infections. The commonest among these clinical conditions was TB. TB and SAM are a major cause of mortality, especially in resource-limited settings for children under the age of five years. The coexistence of both further worsened morbidities and clinical outcomes, with fatality rates reaching up to 56% [17]. Moreover, children under five years have the highest risk of progressing from *Mycobacterium tuberculosis* infection to disease, and to disseminated forms of TB [81]. The relationship between TB and malnutrition exists in a bidirectional manner. Malnutrition heightens susceptibility to active TB by undermining cell-mediated immunity, pivotal for controlling Mycobacterium tuberculosis, or by inciting the reactivation of latent TB infections [82]. On the other hand, TB worsens malnutrition as it causes increased metabolic demands, nutrient malabsorption, and chronic inflammation. Thus, the risk of TB disease increases with undernutrition and TB can cause or worsen undernutrition [75]. One study estimated that 26% of overall TB cases in 22 high-burden countries are attributable to undernutrition [83]. Similarly, a recent review of 51 cohort studies with over 27 million participants from the six WHO regions reported that undernutrition probably increases the risk of TB two-fold in the short term (<10 years) and may also increase the risk in the long term (>10 years) [84]. In contrast, studies included in this review revealed delayed recovery from SAM and high fatality rates in malnourished children with coexisting TB disease compared with cohorts without TB disease [17,29,30].

The high prevalence of TB in malnourished African children can be linked to several socioeconomic factors. Low socioeconomic status, overcrowded living conditions, and food insecurity are common in regions with high rates of SAM and thus contribute to the spread of TB. Additionally, natural or man-made disasters, such as conflicts and displacement, exacerbate food insecurity and poor living conditions, further increasing the risk of TB and other infectious diseases. These factors create an environment where communicable diseases can thrive, especially among individuals with compromised immune systems.

Children afflicted by both TB and SAM frequently present with chronic cough, weight loss, and fever—symptoms that conflate with those of severe malnutrition—thereby rendering diagnosis challenging [81,82]. In addition, it is difficult to make a confirmatory diagnosis using Gene Xpert (Cepheid Inc., Sunnyvale, CA, USA) or cultures as most studies have shown these methods to be unreliable. The diagnosis of TB in the African paediatric population hinges on the ability of the attending physician to make a clinical diagnosis based on presenting symptoms and radiological presentations of the index patient with or without a confirmatory result from the laboratory. Authors’ opined guidelines should be designed for the diagnosis of TB in malnourished children especially in a resource-limited setting where proven diagnostic tools are often limited [16,33].

Besides *Mycobacterium tuberculosis*, other respiratory pathogens such as *Streptococcus pneumoniae* and *Haemophilus influenzae* also contributed to morbidities [36,59], frequently precipitating pneumonia in malnourished children. Consequently, malnourished children often experience protracted illness owing to compromised infection clearance due to weakened respiratory muscles and diminished secretion of protective lung fluids. Furthermore, the weakened immune system in children with SAM amplifies their susceptibility and the gravity of pneumonia. In one study, lower respiratory tract infections were the most common complications second to diarrheal disease, with a frequency of 42.4% (405/956), and were associated with 1.6 times higher odds of dying compared to those who did not have lower respiratory tract infections [19]. Thus, malnutrition induces deficiencies in essential nutrients crucial for a robust immune response, further impeding the body’s ability to combat respiratory infections.

### 4.3. Parasitic Infection

Parasitic infections were also associated with morbidities in severely malnourished children, with malaria parasites accounting for over 90% of the cases. Besides malaria parasites, other parasites including hookworm, *Ascaris lumbricoides*, *Cryptosporidium* species, *Trichuris trichiura*, *Trichomonas intestinalis*, *Entamoeba histolytica*, *Entamoeba dispar*/*histolytica*, *Giardia intestinalis*, *Strongyloides stercoralis*, *Schistosoma haematobium*, *Schistosoma mansoni*, and *Endolimax nana* have also been associated with infections in malnourished children [23,31,85,86]. Although ancylostomiasis (hookworm infection) is common and can cause anaemia, malnutrition, and low protein levels, it is not always a major focus in cases of SAM. This is because the effects of hookworm infections develop slowly, while SAM is usually triggered by more immediate and life-threatening factors like nutritional deficiencies or infections that cause rapid health decline. While hookworm can contribute to long-term malnutrition, it may not be the direct cause of SAM. However, it may be underdiagnosed, making it less frequently reported in SAM cases. Overall, only one study reported a significant correlation between parasite infection and clinical outcomes; a study from Ethiopia aiming to assess the time to recovery from SAM and its predictors reported a high chance of recovery for children who had no anaemia, TB, or malaria infection at admission compared with their counterparts [30].

### 4.4. Fungal Infection

Data on fungal infections in children with SAM were found in only two studies, further affirming the gross neglect of fungal diseases, especially in the paediatric population in Africa. As previously narrated, malnutrition not only results in nutritional deficiencies but also compromises immune function, disrupts the gut microbiota, and alters host defence mechanisms [75]. Consequently, it creates an environment conducive to fungal colonization and infection, heightening the risk for vulnerable populations. Several factors contribute to malnourished children’s increased susceptibility to fungal infections; poor hygiene standards, congested living situations, limited access to clean water and sanitation facilities, and malnutrition-related immunological dysfunction all contribute to fungal colonization and infection in children. In addition, comorbid illnesses like HIV infection raise the risk of fungal infections in malnourished children, emphasizing the importance of integrated healthcare methods that address the complex determinants of health. Despite these myriad factors, the cognizance of fungal infections is still low compared with bacterial and viral infections, as seen in this review. Contrastingly, recent estimates showed that invasive fungal infections have an annual incidence of 6·5 million and account for about 3·8 million deaths globally [87]. This seeming neglect may be accounted for by the sparse data on fungal infections in malnourished children, particularly in the African setting. We recommend prioritizing research investigating malnourished children for invasive mycoses to ascertain the burden of IFIs in this at-risk population, drive awareness of fungal diseases, and decrease morbidity.

### 4.5. Susceptibility Testing of Pathogens

The hallmark of antimicrobial stewardship (AMS) is to ensure the optimized usage of antibiotics and invariably improve clinical outcomes. Its role in preserving and protecting the currently available antibiotics and tackling antimicrobial resistance cannot be over-emphasized. Adherence to AMS strategies implies the indication for antimicrobial therapy, and the antibiotic sensitivity pattern of the associated pathogen is provided and deployed to manage an index case. However, in this review, we identified only three studies reporting the antibiotic susceptibility profile of the associated pathogens (bacteria). This is indicative of the existing gaps regarding antimicrobial usage in a resource-limited setting like Africa and the need to prioritize funding for innovative studies seeking to explore mechanisms or approaches to limit the exposure of malnourished patients to infectious diseases while setting up and ensuring adherence to AMS programmes.

In one of the three studies, the authors reported a high level of resistance to commonly used antibiotics and advocated clinical trials to determine the most feasible combination of antibiotics for managing bacteraemia in severely malnourished children [36]. In another study, the authors suggested increased investment in antibiotic stewardship programmes in the face of increasing rates of drug-resistant bacterial infections among HIV-infected children with SAM [59]. In the other, the authors emphasized the need to tackle the emergence of antibiotic-resistant bacteria by improving diagnostics, ensuring infection control practices, and reinforcing regional antimicrobial resistance surveillance [23].

### 4.6. Clinical Outcomes and Treatment Relapse

Regardless of the setting, whether studies were hospital- or community-based, the treatment outcomes were largely influenced by comorbidities and predominantly of infectious origin [14,15,17,21,22,26,27,30,31,32,34,41,42,51,86]. Regarding treatment relapse, an Ethiopian study reported that the odds of SAM relapse was significantly higher in children with mothers who had no exposure to education and promotion about infant and young child feeding practices, children who were not fully immunized for their age, and children with a mid-upper arm circumference of <12.5 cm at discharge [23]. Similarly, another study reported a lower chance of recovery among children who were not fully vaccinated [29]. Yet in another study, the time to recovery from SAM was delayed in children with comorbidities such as HIV, TB, and pneumonia [39]. The authors recommend the provision of supplementary food for children with a low MUAC at discharge, the promotion of nutrition education, and the improvement in child immunization services and coverage to help reduce SAM relapse [25]. In addition, special emphasis should be given to prevent and treat comorbidities [39].

## 5. Limitations

Some studies reported comorbidities such as pneumonia, diarrhoea, anaemia, gastroenteritis, urinary tract infections, respiratory tract infections, dysentery, meningitis, or sepsis without specifying the associated pathogen, which may have undermined the burden of pathogens identified in this review. Also, the diagnosis of TB was presented in some studies as a clinical diagnosis. A confirmatory diagnosis like the Gene Xpert test or culture or lipoarabinomannan assay was lacking. However, having reviewed a significant number of cases from 60 studies in Africa within the past two decades, we affirm that the findings from this index review can be applied to encourage research on pathogens in African children with SAM, strengthen antimicrobial stewardship programmes in this setting, and invariably decrease morbidity and mortality.

## 6. Conclusions

Ensuring early and accurate diagnosis and treatment of infections in severely malnourished patients is critical to obtain good clinical outcomes. It is crucial to establish community health programmes that emphasize vaccination, hygiene practices, and access to clean water to combat infection, particularly in settings like Africa which are too prone to having children with SAM. Training community health workers to educate families about infection prevention and the early detection of illnesses would be an invaluable tool in driving this [86,88]. In addition, increasing funding for local health facilities to enhance laboratory capabilities and diagnostic tools will facilitate the accurate and rapid identification of infections in malnourished children. This is crucial for the strengthening of healthcare systems, particularly in resource-constrained settings, to ensure that SAM patients receive timely and effective treatment for infections, invariably decreasing morbidity from infections in this at-risk group.

## Figures and Tables

**Table 1:** Summary of original studies reporting pathogens in African children with SAM.

Location/Country	Number of Pathogens/Infection Cases (n)				Fatality Rates (n = Number of Fatal Cases)	No. of Reference
	Viruses	Bacteria	Parasites	Fungi		
^a^ South Africa	HIV (n = 58)	TB (n = 27)	-	-	11.5% (n = 13)	[14]
^b^ Ethiopia		TB (n = 41)	Malaria (n = 37)	-	9.3% (n = 51)	[15]
Sierra Leone	-	TB (n = 20)	-	-	-	[16]
Zambia	-	TB (n = 151)	-	-	56% (n = 84)	[17]
Mozambique	-	TB (n = 17)	-	-	-	[18]
^b^ South Africa	HIV (n = 181)	TB (n = 127)	Malaria (n = 4)	-	25.9% (n = 248)	[19]
Uganda	HIV (n = 76)	-	-	-	-	[20]
^a^ Zambia/ Zimbabwe	HIV (n = 130)	-	-	-	8.5% (n = 55)	[21]
Malawi	HIV (n = 79)	-	-	-	14.8% (n = 67)	[22]
^b^ Niger	Rotavirus (n = 23), Adenovirus (n = 8)	TB (n = 4), bacteraemia (n = 79), enteric pathogens (n = 36), urinary tract pathogens (n = 48)	Malaria parasites (n = 44), intestinal parasites (n = 6)		9% (n = 29)	[23]
^a^ Mozambique	HIV (n = 157)	-	-	-	-	[24]
Ethiopia	HIV (n = 11)	-	-	-	-	[25]
^b^ Ethiopia	-	TB (n = 15)	-	-	7% (NS)	[26]
^b^ South Africa	HIV (n = 23)	TB (n = 17)	-	-	15.1% (n = 19)	[27]
^b^ Ghana	HIV (n = 67)	TB (n = 23), bacteraemia (n = 85)	Malaria (n = 34)	-	17.5% (n = 43)	[28]
^b^ Ethiopia	HIV (n = 1)	TB (n = 27)	Malaria (n = 76)	-	-	[29]
^b^ Ethiopia	HIV (n = 5)	TB (n = 23)	Malaria (n = 2)	-	9% (n = 60)	[30]
Sudan	-	-	Malaria (n = 131), intestinal parasites (n = 24)	-	3.7% (n = 14)	[31]
Kenya	HIV (n = 229)	Bacteraemia (n = 86)	Malaria parasitaemia (n = 227)	-	16% (n = 194)	[32]
Malawi	HIV (n = 52)	TB (n = 2)	-	-	9.7% (n = 29)	[33]
^a,c^ Kenya/Tanzania	-	TB (n = 293)	Malaria (n = 404)	Candidiasis (n = 119)	19% (n = 64) 28% (n = 222)	[34]
Niger	-	TB (n = 90)			19.6% (n = 20)	[35]
^b^ Uganda	HIV (n = 151)	Bacteraemia (n = 76)	-	-	28.9% (n = 22)	[36]
^b^ Ethiopia	HIV (n = 20)	TB (n = 43)	Malaria (n = 7)	-	-	[37]
^b^ South Africa	HIV (n = 196)	-	-	-	24.4% (n = 108)	[38]
^b^ Ethiopia	HIV (n = 54)	TB (n = 107)	-	-	-	[39]
^b^ Democratic Republic of Congo	HIV (n = 14)	Bacteraemia (n = 38)	Malaria (n = 33)	-	9.2% (n = 58)	[40]
^b^ Ethiopia	-	TB (n = 71)	-	-	9% (n = 46)	[41]
^b^ Ethiopia	HIV (n = 21)	TB (n = 16)	Malaria (n = 30)	-	4.4% (n = 9)	[42]
^b^ Ethiopia	-	TB (n = 27)	Malaria (n = 2)	-	16% (NS)	[43]
^b^ Uganda	HIV (n = 20),	TB (n = 17), bacteraemia (n = 23)	Malaria (n = 72)		14.5% (n = 49)	[44]
^b^ Ethiopia	HIV (n = 3)	TB (n = 87)	Malaria (n = 10)	-	10.8% (n = 41)	[45]
^b^ Ethiopia	HIV (n = 5)	-	-	-	11.7% (n = 35)	[46]
^b^ Ethiopia	HIV (n = 17)	TB (n = 9)	Malaria (n = 77)	-	28.7% (n = 119)	[47]
^b^ Ethiopia	HIV (n = 11)	TB (n = 18)	-	-	12.2% (n = 37)	[48]
^b^ Nigeria	HIV (n = 81)	TB (n = 79)	-	-	7.7% (NS)	[49]
^b^ Ethiopia	-	TB (n = 19)	-	-	5.5% (n = 14)	[50]
^b^ Uganda	HIV (n = 43)	-	-	-	9.8% (n = 39)	[51]
^b^ Ethiopia	HIV (n = 26)	TB (n = 37)	Malaria (n = 13)	-	8.5% (n = 34)	[52]
^b^ Cameroon	-	-	Malaria (n = 27)	-	15% (n = 27)	[53]
^b^ Senegal	HIV (n = 6)	TB (n = 2)	-	-	2.9% (n = 3)	[54]
Uganda	-	TB (n = 32)	-	-	-	[55]
^b^ Ethiopia	HIV (n = 9)	TB (n = 17)	Malaria (n = 9)	-	-	[56]
^b^ Ethiopia	HIV (n = 15)	TB (n = 35)	-	-	-	[57]
^b^ Ethiopia	HIV (n = 12)	TB (n = 12)	-	-	2.0% (n = 12)	[58]
^d^ South Africa	HIV (n = 82)	Bacteria (n = 51)	-	-	-	[59]
^b^ Ethiopia	HIV (n = 12)	-	Malaria (n = 9)	-	6.8% (n = 11)	[60]
^b^ Ethiopia	HIV (n = 3)	TB (n = 24)	Malaria (n = 3)	-	3.8% (NS)	[61]
Uganda	HIV (n = 18)	-	Malaria (n = 7)	-	12% (n = 9)	[62]
Ethiopia	HIV (n = 15)	TB (n = 54)	Malaria (n = 21)	-	12.3% (n = 43)	[63]
Malawi	-	TB (n = 4)	-	-	-	[64]
Ethiopia	-	TB (n = 24)	-	-	-	[65]
^b^ Ethiopia	HIV (n = 31)	TB (n = 61)	-	-	11.3% (n = 54)	[66]
Uganda	HIV (n = 9)	-	Malaria (n = 25)	-	-	[67]
Uganda	HIV (n = 33)	-	-	-	25% (n = 67)	[68]
^b^ Uganda	HIV (n = 20)	-	-	-	14% (n = 17)	[69]
^b^ Ethiopia	HIV (n = 3)	TB (n = 43)	-	-	5.9% (NS)	[70]
^b^ Ghana	HIV (54)	TB (n = 32)	Malaria (n = 110)	-	16.5% (n = 99)	[71]
Zambia	HIV (n = 161)	TB (n = 6)	-	-	40.5% (n = 174)	[72]
Uganda	HIV (n = 86)	-	-	-	22% (n = 70)	[73]
**Total**	**2007**	**2275**	**1444**	**119**	**-**	**-**

SAM: severe acute malnutrition, HIV: human immunodeficiency virus, TB: tuberculosis, ^a^: a multicentre study, ^b^: other infections were reported but the associated pathogens were not identified, ^c^: 19% and 28% fatality rates were reported among children admitted at Muhimbili National Hospital in Dar es Salaam, Tanzania, and Kilifi District Hospital in Kenya, respectively, and ^d^: bacteria identified from blood, urine, and sputum cultures. NS: Not stated.

## Data Availability

All underlying data have been included in the manuscript.

## Supplementary Materials

The following supporting information can be downloaded at: https://www.mdpi.com/article/10.3390/tropicalmed9100230/s1, Table S1: Summary of original studies reporting pathogens in malnourished African children.
