# Supplementary material for: The Spectrum of Pathogens Associated with Infections in African Children with Severe Acute Malnutrition: A Scoping Review
Source: Trop Med Infect Dis. 2024 Oct 6;9(10):230. doi: 10.3390/tropicalmed9100230 (PMC11510937; doi:10.3390/tropicalmed9100230)
Supplement: Supplementary file 1 [file tropicalmed-09-00230-s001.zip › tropicalmed-3231036-supplementary.pdf]

Review

The Spectrum of Pathogens Associated with Infections in African Children with Severe Acute Malnutrition: A Scoping Review

Bassey Ekeng [1,*], Olufunke Adedokun [2], Vivien Otu [2], Stella Chukwuma [3], Agatha Okah [4], Osamagbe Asemota [2], Ubokobong Eshiet [2], Usenobong Akpan [5], Rosa Nwagboso [6], Eti Ebiekpi [7], Emmanuella Umoren [8] and Edet Usun [2]

[1] Department of Medical Microbiology and Parasitology, University of Calabar Teaching Hospital, Calabar 540271, Nigeria
[2] Department of Paediatrics, University of Calabar Teaching Hospital, Calabar 540271, Nigeria
[3] Department of Medical Microbiology, College of Medicine, Enugu State University of Science and Technology, Enugu 400283, Nigeria
[4] Department of Paediatrics, University Hospitals Coventry and Warwickshire, NHS Trust, Coventry CV2 2DX, UK
[5] Department of Paediatrics, University of Uyo Teaching Hospital, Uyo 520261, Nigeria
[6] Department of Family Medicine, University of Calabar Teaching Hospital, Calabar 540271, Nigeria
[7] Department of Community Medicine, University of Uyo Teaching Hospital, Uyo 520261, Nigeria
[8] Department of Paediatrics, Leeds Teaching Hospital Trust, Leeds LS1 3EX, UK

* Correspondence: basseyewaekenggmail.com; Tel.: +2348037361816

Supplementary Table S1. Summary of original studies reporting pathogens in malnourished African children.

Location/ Country	Type of Study	HB/ OTP	Study period	Study population	Sample size (n)	Age range or Median age	Number of Pathogens / Infection cases (n)	Fatality rates (n=number of fatal cases)	Authors/Year of publication	No. of reference
[a]South Africa	Prospective, observational study	HB	NS	SAM	113	< 5 years	HIV (n=58), TB (n=27)	11.5% (n=13)	De Maayer et al 2011	14
[b]Ethiopia	Retrospective cohort study	HB	2013 to 2015	SAM	545	< 5 years	Malaria (n=37), TB (n=41)	9.3% (n=51)	Girum et al 2017	15
Sierra Leone	Descriptive cross-sectional study	HB	Over 6 months in 2018	SAM	74	Median age of 11 months	TB (n=20)	-	Ide et al, 2019	16

Zambia	Retrospective study	HB	2009 to 2013	SAM	9,450	0 to 59 months	TB (n=151)	56% (n=84)	Munthali et al 2017	17.
Mozambique	Retrospective study	HB	February to August 2018	SAM	45	0 to 59 months	TB (n=17)	-	Osorio et al. 2020	18
[b]South Africa	Cross-sectional study	HB	2014 – 2018	SAM	956	Children under 5 years	HIV (n=181) TB (n=127) Malaria (n=4)	25.9% (n=248)	Gavhi et al.2019(2020)	19
Uganda	Cross-sectional study	HB	June 2021 to December 2022	SAM	797	1 month to 5 years	HIV (n=76)	-	Musiime et al. 2024	20
[a]Zambia/ Zimbabwe	Prospective cohort study	HB	July 2016 and March 2019	SAM	649	1 month to 59 months	HIV (n=130)	8.5% (n=55)	Bwakura-Dangarembizi et al, 2021	21
Malawi	Prospective cohort study	HB	NS	SAM	454	6 months to 59 months	HIV (n=79)	14.8% (n=67)	Chinkhumba et al, 2008	22
[b]Niger	Prospective study	HB	November 2007 to July 2008	SAM	311	6 months to 59 months	Bacteremia, n=79, malaria parasite, n=44, enteric pathogens isolated from stool (bacteria, n=36, viruses, n=23, intestinal parasites, n=6), TB (n=4), pathogens isolated from the urinary tract (bacteria, n=48), pathogens identified from nasal swabs (viruses, n=5)	9% (n=29)	Page et al, 2013	23
Mozambique	Retrospective observational study	HB	March 2016 to February 2017	SAM	1,231	0 to 5 years	HIV (n=157)	-	Calgaro et al, 2021	24
Ethiopia	Cross-sectional study	HB	April – June 2020	SAM	208	6 months to 59 months	HIV (n=11)	-	Teshale et al, 2023	25

[b]Ethiopia	Retrospective cohort study	HB	January 2012 to December 2015	SAM	500	Under 5 years	TB (n=15)	7% (NS)	Yohannes et al, 2017	26
[b]South Africa	Retrospective cohort study	HB	October 2014 to December 2018.	SAM	126	0 to 59 months	HIV (n=23), TB (n=17)	15.1 % (n=19)	Heydenrych et al, 2024	27
[b]Ghana	Cross-sectional prospective study	HB	February 2010 to October 2010	SAM	246	3 months to 13 years	HIV (n=67), TB (n=23), malaria (n=34), bacteremia (n=85)	17.5% (n=43)	Asafo-Agyei et al 2013	28
[b]Ethiopia	Retrospective follow-up study	HB	March to April, 2018	SAM	398	6 months to 59 months	HIV (n=1), malaria (n=76), TB (n=27)	-	Wondim et al, 2020	29
[b]Ethiopia	Retrospective cohort study	HB	September 2017 to March 2020.	SAM	665	0 to 59 months	HIV (n=5), TB (n=23), malaria (n=2)	9% (n=60)	Oumer et al, 2021	30
Sudan	Prospective hospital-based study	HB	April to October 2018	SAM	376	6 months to 59 months	Malaria (n=131), intestinal parasites (n=24)	3.7% (n=14)	Bilal et al, 2020	31
Kenya	Prospective descriptive study	HB	June 2005 to June 2009	SAM	1,206	6 months to 12 years	HIV (n=229), malaria parasitemia (n=227), bacteremia (n=86)	16% (n=194)	Talbert et al, 2012	32
Malawi	Cross-sectional observational study	HB	February to May 2012	SAM	300	6 months to 60 months	HIV (n=52), TB (n=2)	9.7% (n=29)	LaCourse et al. 2014	33
[a,c]Kenya/Tanzania	A retrospective study	HB	2004 to 2005	SAM	1121	NS	Malaria (n=404), candidiasis (n=119), TB (n=293)	19% (n=64) 28% (n=222)	Sunguya et al, 2006	34
Niger	Cross-sectional study	HB	2016 to 2017	SAM	202	< 5 years	TB (n=90)	19.6% (n=20)	Schramm et al. 2021	35
[b]Uganda	Prospective study	HB	September-November 2003 and September-	SAM	450	<60 months	HIV (n=151), bacteremia (n=76)	28.9% (n=22)	Bachou et al, 2006	36

			December 2004							
[b]Ethiopia	Retrospective cross-sectional study	HB	2018 – 2020	SAM	414	< 5 years	Malaria (n=7), HIV (n=20), TB (n=43)	-	Atalell et al, 2021	37
[b]South Africa	A retrospective multicohort study	HB	2009 – 2013	SAM	454	6 months to 60 months	HIV (n=196)	24.4% (n=108)	Muzigaba et al, 2017	38
[b]Ethiopia	Retrospective	HB	2012 - 2016	SAM	1690	The majority of the participants were < 2 years	TB (n=107), HIV (n=54)	-	Baraki et al, 2020	39
[b]Democratic Republic of Congo	Retrospective	HB	2017 - 2018	SAM	633	1 month to 18 years	HIV (n=14), malaria (n=33), bacteremia (n=38)	9.2% (n=58)	Kambale et al, 2020	40
[b]Ethiopia	Retrospective cohort study	HB	2012 – 2019, (may to June 2019)	SAM	515	Majority were < 24 months	TB (n=71)	9% (n=46)	Bitew et al, 2020	41
[b]Ethiopia	Retrospective cross-sectional study	HB	2015 - 2017	SAM	205	1 month – 14 years	HIV (n=21), TB (n=16), malaria (n=30)	4.4% (n=9)	Mena et al, 2018	42
[b]Ethiopia	Retrospective study	HB	2013 - 2015	SAM	196	Median age: 12+8.5 months	TB (n=27), malaria (n=2)	16% (NS)	Kabeta et al, 2017	43
[b]Uganda	Analytical and Descriptive Prospective Cohort Study	HB	July to September 2019	SAM	338	< 5 years	Malaria (n=72), bacteremia (n=23), HIV (n=20), TB (n=17)	14.5% (n=49)	Banga et al, 2020	44
[b]Ethiopia	Retrospective cohort study	HB	2015 to 2017	SAM	420	6 months to 59 months	HIV (n=3), TB (n=87), malaria (n=10)	10.8% (n=41)	Fikrie et al, 2019	45
[b]Ethiopia	Cross-sectional study	HB	2010 to 2012	SAM	298	2 months to 59 months	HIV (n=5)	11.7% (n=35)	Abeje et al 2016	46
[b]Ethiopia	A Retrospective Cohort Study	HB	2011 to 2013	SAM	415	0 to 59 months	TB (n=9), HIV (n=17), malaria (n=77)	28.7% (n=119)	Desta et al, 2015	47
[b]Ethiopia	A Retrospective Cohort Study	HB	2013 to 2016	SAM	259	6 months to 59 months	TB (n=18), HIV (n=11)	12.2% (n=37)	Negussie et al, 2020	48

[b]Nigeria	Prospective cohort study	HB	2017 to 2019	SAM	100	Mean age: 14.28 ± 14.04 months	HIV (n=81), TB (n=79)	7.7% (NS)	Ikobah et al, 2022	49
[b]Ethiopia	Retrospective cohort study	HB	2014 to 2016	SAM	253	6 months to 59 months	TB (n=19)	5.5% (n=14)	Mekuria et al, 2017	50
[b]Uganda	Prospective cohort study	HB	2014 to 2015	SAM	400	6 months to 59 months	HIV (n=43)	9.8% (n=39)	Nabukeera-Barungi et al, 2017	51
[b]Ethiopia	Cross-sectional study	HB	2012 to 2016	SAM	401	6 months to 59 months	TB (n=37), HIV (n=26), malaria (n=13)	8.5% (n=34)	Desyibelew et al 2017	52
[b]Cameroon	Retrospective study	HB	2006 to 2015	SAM	179	< 15 years	Malaria (n=27)	15% (n=27)	Chiabi et al, 2016	53
[b]Senegal	Descriptive and analytical cross-sectional study	HB	March to November, 2021	SAM	103	6 months to 59 months	TB (n=2), HIV (n=6)	2.9% (n=3)	Ba et al, 2023	54
Uganda	Cross-sectional study	HB	2023 - 2024	SAM	137	6-59 months	TB (n=32)	-	Asiimwe et al, 2024	55
[b]Ethiopia	Cross-sectional study	HB	Not stated	SAM	351	0.5-14 years	HIV (n=9), TB (n=17), malaria (n=9)	-	Girma et al 2013	56
[b]Ethiopia	Retrospective study	HB	2015 - 2019	SAM	454	6 months to 59 months	HIV (n=15), TB (n=35)	-	Bizuneh et al 2022	57
[b]Ethiopia	Retrospective cohort study	OTP	2016 - 2019	SAM	600	Birth to 59 months	HIV (n=12), TB (n=12)	2.0% (n=12)	Abate et al 2020	58
[d]South Africa	Prospective	HB	2012 - 2015	SAM	82	1 month to 10.6 years	HIV (n=82), Bacteria (n=51)	-	Archary et al, 2016	59
[b]Ethiopia	A retrospective cohort study	HB	January to February, 2021	SAM	162	6 months to 59 months	Malaria (n=9), HIV (n=12)	6.8% (n=11)	Aye et al, 2023	60
[b]Ethiopia	A prospective cohort study	HB	March to July, 2018	SAM	133	6 months to 59 months	TB (n=24), HIV (n=3), malaria (n=3)	3.8% (NS)	Adem et al, 2020	61
Uganda	A prospective cohort study	HB	2010 - 2011	SAM	74	6 months – 5 years	HIV (n=18), malaria (n=7)	12% (n=9)	Mody et all, 2014	62
Ethiopia	A Retrospective Cohort Study	HB	2015 – 2017	SAM	375	6 months to 59 months	HIV (n=15), TB (n=54), malaria (n=21)	12.3% (n=43)	Kabthymer et al, 2020	63
Malawi	Prospective observational study	HB	2021 - 2022	SAM	131	6 months to 59 months	TB (n=4)	-	Vonasek et al, 2024	64

Ethiopia	Retrospective cohort study	HB	2018 - 2022	SAM	247	< 5 years	TB (n=24)	-	Wake et al, 2024	65
[b]Ethiopia	Retrospective, Cohort study	HB	2016 to 2019	SAM	476	< 5 years	HIV (n=31) TB (n=61)	11.3% (n=54)	Kassaw et al 2021	66
Uganda	Prospective cohort study	HB	June to August 2015	SAM	122	Children under 5 years	HIV (n=9) Malaria (n=25)	-	Nduhukire et al.2020	67
Uganda	Prospective cohort study	HB	November 2007 to July 2008	SAM	270	<5yrs	HIV (n=33)	25% (n=67)	Nwalanga et al 2020	68
[b]Uganda	Prospective observational study	HB	2012 - 2013	SAM	120	6 months to 59 months	HIV (n=20)	14% (n=17)	Rytter et al, 2017	69
[b]Ethiopia	Retrospective cohort study	HB	December 10-30, 2021	SAM	712	6 months to 59 months	TB (n=43), HIV (n=3)	5.9% (NS)	Ahmed et al, 2023	70
[b]Ghana	Prospective observational study	HB	2013 to 2018	SAM	601	0 to 59 months	HIV (54), TB (n=32), malaria(n=110)	16.5% (n=99)	Asare et al, 2021	71
Zambia	Cohort study	HB	August - December 2009	SAM	430	6 months to 59 months	HIV (n=161) TB (n=6)	40.5% (n=174)	Irena et al 2013	72
Uganda	Retrospective observational study	HB	January to December 2017	SAM	330	1-5yrs	HIV (n=86)	22% (n=70)	Muwanguzi et al, 2021	73

SAM: Severe acute malnutrition, HIV: Human immunodeficiency virus, UTI: Urinary tract infection, TB: Tuberculosis, HB: Hospital-based study, OTP: Outpatient therapeutic programme, NS: Not stated, a: A multicenter study, b: Other infections were reported but the associated pathogens were not identified, c: 19% and 28% fatality rates were reported from children admitted among children admitted at Muhimbili National Hospital in Dar es Salaam, Tanzania and Kilifi District Hospital in Kenya, respectively, d: Bacteria identified from blood, urine and sputum culture
